# Use of chiral cell shape to ensure highly directional swimming in trypanosomes

**DOI:** 10.1371/journal.pcbi.1005353

**Published:** 2017-01-31

**Authors:** Richard John Wheeler

**Affiliations:** 1 Sir William Dunn School of Pathology, University of Oxford, Oxford, United Kingdom; 2 Max Planck Institute of Molecular Cell Biology and Genetics, Dresden, Germany; University of Illinois at Urbana-Champaign, UNITED STATES

## Abstract

Swimming cells typically move along a helical path or undergo longitudinal rotation as they swim, arising from chiral asymmetry in hydrodynamic drag or propulsion bending the swimming path into a helix. Helical paths are beneficial for some forms of chemotaxis, but why asymmetric shape is so prevalent when a symmetric shape would also allow highly directional swimming is unclear. Here, I analyse the swimming of the insect life cycle stages of two human parasites; *Trypanosoma brucei* and *Leishmania mexicana*. This showed quantitatively how chirality in *T*. *brucei* cell shape confers highly directional swimming. High speed videomicrographs showed that *T*. *brucei*, *L*. *mexicana* and a *T*. *brucei* RNAi morphology mutant have a range of shape asymmetries, from wild-type *T*. *brucei* (highly chiral) to *L*. *mexicana* (near-axial symmetry). The chiral cells underwent longitudinal rotation while swimming, with more rapid longitudinal rotation correlating with swimming path directionality. Simulation indicated hydrodynamic drag on the chiral cell shape caused rotation, and the predicted geometry of the resulting swimming path matched the directionality of the observed swimming paths. This simulation of swimming path geometry showed that highly chiral cell shape is a robust mechanism through which microscale swimmers can achieve highly directional swimming at low Reynolds number. It is insensitive to random variation in shape or propulsion (biological noise). Highly symmetric cell shape can give highly directional swimming but is at risk of giving futile circular swimming paths in the presence of biological noise. This suggests the chiral *T*. *brucei* cell shape (associated with the lateral attachment of the flagellum) may be an adaptation associated with the bloodstream-inhabiting lifestyle of this parasite for robust highly directional swimming. It also provides a plausible general explanation for why swimming cells tend to have strong asymmetries in cell shape or propulsion.

## Author summary

Swimming cells often follow a helical swimming path, however the advantage of helical paths over a simple straight line path is not clear. To analyse this phenomenon, I analysed the swimming of the human parasites *Trypanosoma brucei* (which causes sleeping sickness/trypanosomiasis) and *Leishmania mexicana* (which causes leishmaniasis). Using new computational methods to determine the three dimensional shape of swimming cells I showed that *T*. *brucei* have a helical shape which causes rotation as the cell swims, and the geometry of the resulting swimming path makes the cell movement highly directional. In contrast, *L*. *mexicana* are symmetrical, do not rotate, and their swimming paths are curved and have low directionality. Using a *T*. *brucei* mutant I showed that the cell structure responsible for the helical shape while swimming is the flagellum attachment zone. This explains a key function of this structure. Finally, simulations showed the phenomenon of rotation while swimming is a way cells can ensure highly directional swimming along a controlled helical path, overcoming random variation in cell shape or propulsion. This provides a general explanation for why swimming cells are often asymmetric and tend to follow helical paths.

## Introduction

Many swimming microorganisms and cells swim along helical paths; examples come from all scales of microscopic life, including the multicellular zooplankton *Ploesoma*[1], ciliates like *Paramecium*[2], and bacteria like *Caulobacter*[3]. This has been recognised for over a century[1], and a helical path (ignoring the effects of Brownian motion) is the default trajectory for a swimming cell at low Reynolds number[4,5]. Helicity of the path arises from chiral asymmetry in cell shape (and so hydrodynamic drag) or propulsion mechanism[4,5] and is the basis of the deterministic chemotaxis mechanism ‘helical clinotaxis’[6,7]. In the absence of a chemotactic (or similar) stimulus a helical path confers directionality along the axis of the helix. Famous examples, across the scales of microscopic life, include the multicellular *Volvox*[8], the unicellular alga *Chlamydomonas*[9] and the bacterium *Bacillus subtilis*[10]. This behaviour has been referred to as ‘spin stabilisation’[1], but is distinct from conventional rotational inertia-based (gyroscopic) spin stabilisation which is not possible in low Reynolds number environments where inertia is negligible.

Swimming along a straight line is a beneficial behaviour for chemotaxis, either to maximise displacement during run and tumble chemotaxis or to travel directly up a chemoattractant gradient in helical clinotaxis. The naïve approach to achieving straight line swimming is perfect symmetry in propulsion and cell shape, as an asymmetry in either could bend a straight swimming path into a futile circular path. However, cells which can achieve highly directional, near straight line, swimming tend to have chirally asymmetric propulsion or cell shape[1–3,8–10]. The human parasite *Trypanosoma brucei* (which causes sleeping sickness/African trypanosomiasis) is a characteristic example of this. It has a strongly asymmetric cell organisation with its single flagellum laterally attached to the cell body for much of its length by the flagellum attachment zone (FAZ)[11,12], and is capable of achieving highly directional swimming in both the fly gut-inhabiting (procyclic) and mammalian bloodstream-inhabiting (bloodstream) life cycle stages[13–17]. Motility is critical for the bloodstream form[18,19], and the parasite is well adapted for motility among erythrocytes[16,20]. The general fact that swimming microorganisms seem to favour chiral asymmetric propulsion and shape over symmetry for achieving highly directional swimming is recognised[5], but not rigorously analysed.

*T*. *brucei* is a member of a family of parasites which also includes *Leishmania mexicana* (which causes human and animal leishmaniasis). *T*. *brucei* and *L*. *mexicana* share the typical features of trypanosomatid cells; a single flagellum and long, thin, cell body whose shape is defined by a parallel array of sub-pellicular microtubules[12]. They achieve morphological diversity within this framework; where the flagellum exits the cell body, and which portion of the flagellum lies laterally attached to the cell body[21]. Their morphology is precisely replicated during division events[22–25] and precisely altered during life cycle stage differentiation events[26–28]. *T*. *brucei* and *L*. *mexicana* have differing asymmetry in cell shape; *Leishmania* morphogenesis and cell shape are more symmetric[23,29]. All trypanosomatid morphological classes, except the amastigote, are motile using a tip to base flagellum beat for swimming[13,16,30,31]. The swimming of both of these parasites is important for the normal life cycle[32–34].

Through analysis of these two human parasites, including a *T*. *brucei* morphological mutant with a shorter FAZ[35], I showed capacity for highly directional swimming correlated with longitudinal rotation rate while swimming. Fitting of an analytical model of cell movement to high speed videomicrographs showed that *T*. *brucei* cells have a helical shape (distorted by flagellum movement) while *L*. *mexicana* do not. The capacity for longitudinal rotation correlated with calculated torque from hydrodynamic drag under constant fluid flow due to helicity of cell shape. The *T*. *brucei* morphological mutant showed helical cell shape is conferred by the lateral attachment of the *T*. *brucei* to the cell body, suggesting the function of the extended flagellum attachment zone (FAZ) may be to generate helicity in cell shape and confer the ability for more robust directional swimming. As this extended FAZ is an adaptation associated with the bloodstream-inhabiting lifestyle of *Trypanosoma* parasites[21], this may be a necessary adaptation for the bloodstream environment.

Simulation of cell swimming with longitudinal rotation confirmed that rotation rate is sufficient to explain the large-scale swimming behaviours: ‘Spin stabilisation’ arose through the helical geometry of the resulting swimming path, and straight line swimming of *T*. *brucei* arose from a long pitch, low radius, helical path. This simulation also quantitatively demonstrated that this spin stabilised swimming is tolerant of small variations in either shape or propulsion (biological noise). However, cells which achieve highly directional swimming through avoiding asymmetry are not. This suggests that the commonality of chiral asymmetric shape and propulsion in swimming cells and microorganisms is to allow highly directional swimming in the presence of biological noise in cell shape and propulsion.

## Results

The *Leishmania mexicana* promastigote and *Trypanosoma brucei* procyclic trypomastigote are of similar size, both normally inhabit a similar (fly gut) environment, and both are grown at the same temperature (28°C). However, they have differences in morphology. Additionally, a viable morphological mutant of *T*. *brucei* procyclic trypomastigotes (ablation of ClpGM6, Tb927.11.1090, by RNAi) has recently been described with a shortened FAZ which gives an intermediate (epimastigote-like) morphology[35]. This allows the analysis of swimming of a range of trypanosomatid morphologies under the same conditions.

### Different morphologies of trypanosomatid have different swimming behaviours

To determine if the swimming of promastigote *L*. *mexicana* and procyclic form (trypomastigote) *T*. *brucei* differ, their swimming was analysed using low magnification dark field videomicrographs. Videomicrographs were captured from parasites swimming in 0.25 mm deep chambers to minimise any surface entrainment effect. Cells were detected and swimming tracks were determined using automated tools written in the ImageJ macro language[36] (see Methods). Accuracy and effectiveness of this image analysis method to detect cells at different distances from the focal plane was assayed using a focus stack of *L*. *mexicana* cells adhered to a glass slide (instead of being free to swim). Cells could be reliably detected by this method within approximately 10 μm of the focal plane, with the proportion detected dropping exponentially beyond that range (S1A Fig). The tracks are therefore “optically confined” to a depth of approximately 10 μm either side of the focal plane so represent a two dimensional projection of three dimensional swimming paths in this volume. The automated image analysis yielded cell swimming paths with a mean length of around 30 s, typically with 300 paths over 5 s in length per 512 frame video (S1B Fig).

**Fig 1:**
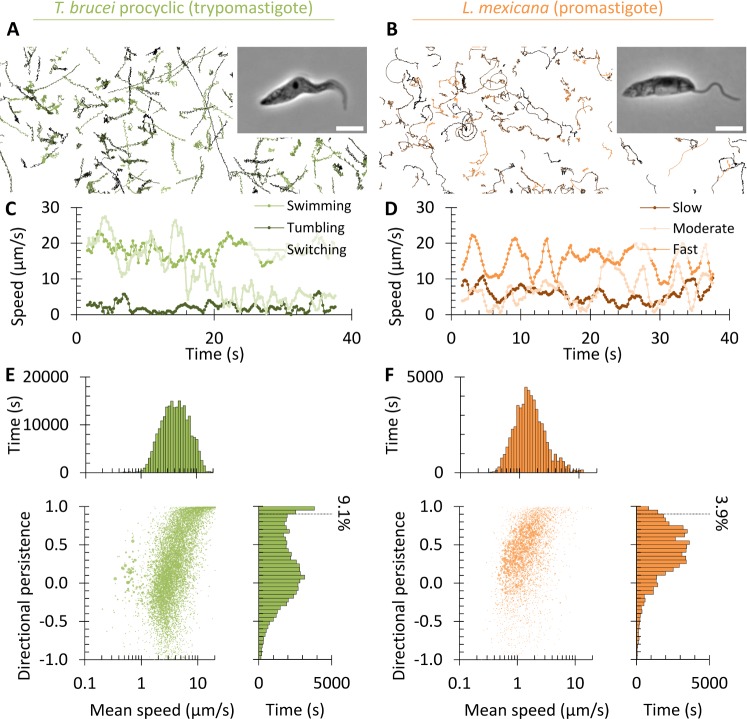
Swimming behaviours of trypomastigote *T*. *brucei* procyclic forms and promastigote *L*. *mexicana* promastigotes have characteristic differences. **(A,B)** Paths followed by swimming trypomastigote *T*. *brucei* procyclic forms (**A**) and promastigote *L*. *mexicana* (**B**), determined from low magnification dark field videomicrographs captured at 5 Hz. Trypomastigotes underwent fast swimming or tumbling, while promastigotes followed curved paths. An example phase contrast image of cell morphology is shown as an inset (scale bar represents 5 μm). **(C,D)** Swimming speed over time of an example swimming, tumbling and switching trypomastigote *T*. *brucei* procyclic form (**C**) and an example fast, moderate and slow swimming promastigote *L*. *mexicana* (**D**). **(E,F)** Distributions and cross correlation of swimming velocity and directional persistence for trypomastigote *T*. *brucei* procyclic form (**E**), promastigote *L*. *mexicana* (**F**). The percentage of swimming with directional persistence >0.9 is indicated. Data was pooled from at least three biological replicates with at least 4,000 cell tracks. Point area in the scatter plots is proportional to the length of the cell track to which it corresponds.

Analysis of the swimming tracks showed *T*. *brucei* trypomastigotes and *L*. *mexicana* promastigotes swim at a similar speed. *T*. *brucei* trypomastigotes switched between fast highly directional swimming and slow tumbling behaviours (similar to the bloodstream form[14,15]), while *L*. *mexicana* promastigotes swam at a variable speed along curving tracks with occasional sharp changes in direction (Fig 1A–1D). *T*. *brucei* tumbling and *L*. *mexicana* direction changes are likely related phenomena associated with a reversal of flagellum beat direction[13,14,37]. The behaviours while swimming were quantified by treating the noisy track data as a biased random walk (as previously used to analyse bloodstream form *T*. *brucei*[17]). Two statistics were calculated from swimming tracks, average speed and directional persistence over an analysis interval of 2 s (S1C Fig). Tracks were weighted by their length in seconds (see Methods). Average directional persistence is the average cosine of the change in direction of travel between time steps, a measure of directionality of swimming from oscillating movement (-1) through non-directional movement (0) to highly directional movement (1). It is a measure of directionality not dependent on track length, and is relatively insensitive to occasional sharp changes in direction. This showed two distinct classes of behaviour for *T*. *brucei* trypomastigotes; tumbling (low speed, low directionality) and directional swimming (high speed, very high directionality). There was one class of behaviour for *L*. *mexicana* promastigotes; swimming (moderate speed, moderate directionality) (Fig 1E and 1F). Within each of these classes there was some variation, giving broad distributions of swimming speed and persistence of individual cells.

Given these differences in swimming I next considered two hypotheses as to how the differences may arise: (i) From the differences between the promastigote and trypomastigote morphology, or (ii) from differences in the flagellum beat structure and regulation between these two species.

If the cell morphology is responsible for the differences in swimming behaviours then alteration of *T*. *brucei* procyclic trypomastigote morphology to increase similarity to the promastigote may alter the swimming behaviours to be more similar to the promastigote. This was tested using the *T*. *brucei* tetracycline inducible ClpGM6 RNAi cell line[35]. The trypomastigote flagellum is laterally attached to the cell by a long flagellum attachment zone (FAZ) (17.7±2.6 μm, n = 50), and depletion of ClpGM6 for 72 h causes a shortening of the FAZ (6.6±2.0 μm, n = 50). This causes alteration of the cell from a trypomastigote morphology with a short length of free flagellum (3.4±1.1 μm, n = 50) to an epimastigote-like shape with a long free portion of flagellum (13.9±2.5 μm, n = 50), more similar to a promastigote. Induction of ClpGM6 RNAi has little effect on cell growth relative to the uninduced cell line or the parental 29–13 cell line[35], although 29–13 cells (which express T7 polymerase and Tet repressor to allow inducible RNAi[38]) grow more slowly and have slightly altered morphology in comparison to wild-type trypanosomes[39].

**Fig 2:**
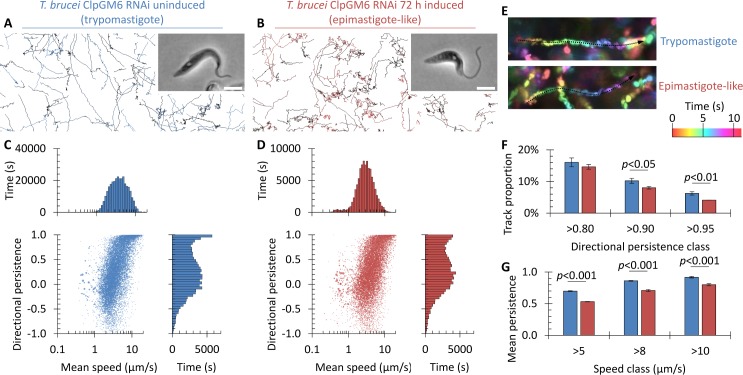
Swimming of procyclic *T*. *brucei* is subtly altered by shortening of the FAZ and change to an epimastigote-like morphology. **(A,B)** Paths followed by the *T*. *brucei* ClpGM6 RNAi cell line before induction (trypomastigote) (**A**) and 72 h after induction (epimastigote-like) (**B**). An example phase contrast image of cell morphology is shown as an inset (scale bar represents 5 μm). **(C,D)** Distribution and cross correlation of swimming velocity and directional persistence for the ClpGM6 RNAi cell line before (**C**) and after (**D**) induction, presented as in Fig 1E and 1F, showing a change in the most highly directional swimming. Data was pooled from three biological replicates with at least 10,000 cell tracks. Point area in the scatter plots is proportional to the length of the cell track to which it corresponds. **(E)** Colour-coded maximum projections of example fast-swimming trypomastigote and epimastigote-like cells illustrating typical swimming paths. Epimastigote-like swimming paths tended to be more curved. **(F)** Percentage of tracks over 0.80, 0.90 or 0.95 persistence for trypomastigote and epimastigote-like cells. Highly directional paths were more common for the trypomastigote. Error bars indicate standard deviation of three biological replicates, significant differences (Student’s t-test) are indicated. **(G)** Mean persistence of tracks over 5, 8, or 10 μm/s for trypomastigote and epimastigote-like cells. Fast swimming trypomastigotes cells were more directional. Error bars indicate standard deviation of three biological replicates, significant differences (Student’s t-test) are indicated.

Quantitative analysis of swimming behaviours before induction of ClpGM6 RNAi showed behaviours overall similar to wild-type *T*. *brucei* (Fig 1A and 1E and Fig 2A and 2C). 72 h after induction of ClpGM6 RNAi there was with no significant change in average swimming speed (4.80±0.37 to 5.20±0.23 μm/s, n = 3, Student’s t-test *p*>0.1) or average directional persistence (0.289±0.027 to 0.303±0.016, n = 3, Student’s t-test *p*>0.4), however there was a significant change (KS test, *p*<0.01) in directional persistence distribution (Fig 2B and 2I). This change was a reduction in the number of cells undergoing highly directional motion, visible as more curved swimming tracks (Fig 2E). The proportion of highly directional tracks (>0.90 and >0.95 directional persistence) significantly decreased following ClpGM6 RNAi (Fig 2F). Furthermore, the mean persistence of fast swimming cells (>5, >8 or >10 μm/s, so excluding tumbling cells) was significantly decreased following ClpGM6 RNAi (Fig 2G). Shortening of the FAZ and increase in the free flagellum length therefore has an effect on one specific aspect of the swimming behaviour of a trypomastigote; to reduce the capacity for highly directional swimming. This alters the swimming behaviour to be more similar to a promastigote, suggesting the cell morphology is responsible for the differing behaviours.

### Differences in swimming behaviours correlate with longitudinal rotation rate

**Fig 3:**
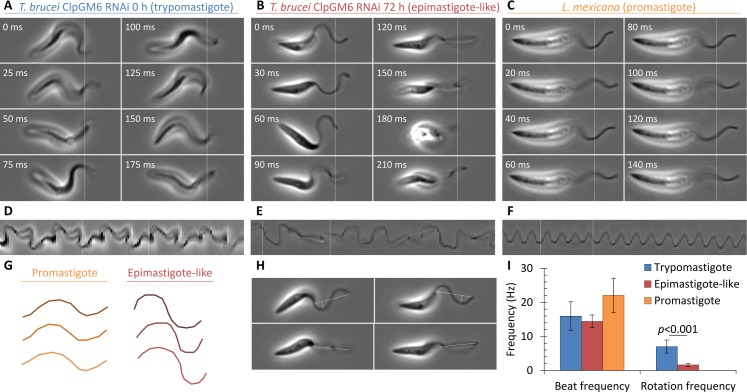
Flagellum beat and cell shape changes in the swimming motion of promastigote, trypomastigote and epimastigote-like cells. **(A-C)** Stills from high magnification high frame rate videomicrographs of swimming uninduced (trypomastigote) ClpGM6 RNAi *T*. *brucei* procyclic form cell line (**A**), 72 h induced (epimastigote-like) ClpGM6 RNAi (**B**) and promastigote *L*. *mexicana* (**C**), captured at 200 Hz. Cell translation from swimming was digitally subtracted. Time through the videomicrograph is shown in the top left, the dotted line shows the fixed position from which the kymographs in (**D-F**) were generated. **(D-F)** Kymographs of apparent flagellar motion through a transverse section through the flagellum, as indicated in (**A-C**). Non-sinusoid traces indicate a deviation from a planar sinusoid-like flagellum beat through beat movement in three dimensions or cell rotation. The dotted lines indicate the first and last stills in (**A-C**). **(G)** Three representative traces of the flagellum beat shape of promastigote *L*. *mexicana* and epimastigote-like *T*. *brucei* cells. **(H)** Examples of the ratio of maximum (orthogonal) to minimum (parallel) apparent flagellum beat amplitude as longitudinal rotation of epimastigote-like *T*. *brucei* cells provided views orthogonal and parallel to the plane of flagellum beat. (**I**) Mean flagellum and cell rotation frequencies of promastigote *L*. *mexicana*, trypomastigote *T*. *brucei* and epimastigote-like *T*. *brucei*. Error bars represent standard deviation, significance of the change in rotation rate between the trypomastigote and epimastigote-like morphology (Student’s t-test) is indicated.

Previous work suggests there may be differences in flagellum beat structure between the *T*. *brucei* procyclic trypomastigote and the *L*. *mexicana* promastigote which may be responsible for differences in swimming behaviours[13,31,40]. However there has not been analysis of flagellum beat shape in *T*. *brucei* in the absence of the effects of lateral attachment of the flagellum to the cell body. Therefore high frame rate videomicrographs between 0.9 and 3.0 s long were used to analyse the flagellum beat in *T*. *brucei* procyclic form before and after depletion of ClpGM6 by RNAi in comparison to *L*. *mexicana* procyclic forms (Fig 3A–3F, S1 Video). Average cell motion over the course of the videomicrograph was subtracted, to keep the cell at a constant position, and kymographs were generated at a fixed position through the cell (Fig 3D–3F). 72 h after induction of ClpGM6 RNAi the resulting epimastigote-like cells underwent longitudinal rotation while swimming (similar to existing descriptions of the *T*. *brucei* bloodstream form trypomastigote[16]) but *L*. *mexicana* promastigotes did not (see below). The flagellum beat was similar, with a canonical sinusoid-like shape in both cell types, but a larger amplitude in the epimastigote-like (Fig 3B, 3C and 3G). When the epimastigote-like cells rotated such that the plane of the flagellar beat was orthogonal to the focal plane no deviation from a planar beat structure could be seen (Fig 3H). Equivalently, this appears as a periodic drop in apparent beat amplitude to near-zero in kymographs of the flagellar beat (Fig 3E). Therefore, in the absence of the effects of lateral cell body attachment, the *T*. *brucei* and *L*. *mexicana* flagellar beats are very similar. As they both appear planar they cannot generate a rotational moment to drive longitudinal rotation and be directly responsible for the differing swimming behaviours. Instead cell shape is presumably responsible. This also indicates that the *T*. *brucei* flagellum does not have an intrinsic ability to undergo a non-planar flagellar beat, incompatible with the proposal that the paraflagellar rod introduces helicity to give a bihelical beat[31,40].

The striking difference in cell movement while swimming between epimastigote-like *T*. *brucei* and *L*. *mexicana* promastigotes was occurrence of longitudinal rotation. Using high magnification high frame rate videomicrographs, rotation of trypomastigote (uninduced ClpGM6 RNAi), epimastigote-like (72 h ClpGM6 RNAi) and promastigote (*L*. *mexicana*) cells while swimming was analysed. Rotation was identified from changes in orientation of the posterior of the cell each time a new flagellum waveform initiated from the anterior flagellum tip. Number of cell rotations or flagellar beats over the length of the videomicrograph was used to calculate beat and rotation frequency. The reorientation of the cell posterior of the trypomastigote did not appear oscillatory, unlike previously described for the bihelical beat model of procyclic form motility[31]. The frequency of the flagellar beat was similar for all three (Fig 3I), however occurrence of longitudinal rotation differed: *L*. *mexicana* did not rotate around their longitudinal axis on this time scale (*n* = 6), while both induced and uninduced ClpGM6 RNAi did (*n* = 8 and *n* = 7 respectively). Rate of longitudinal rotation was significantly reduced (t-test, *p*<0.001) following ClpGM6 RNAi (Fig 3I). The coarse correlation of longitudinal rotation with swimming behaviour supports the view that longitudinal rotation is the aspect of cell movement responsible for the difference in swimming behaviours.

### Simulation shows differences in directionality can be accounted for by differences in longitudinal rotation

**Fig 4:**
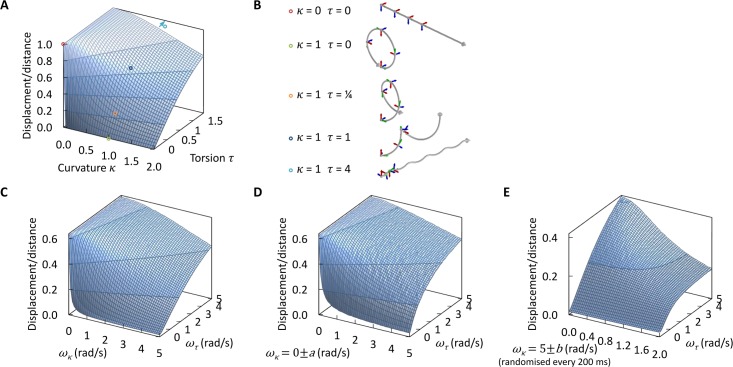
Swimming path curvature and torsion define swimming directionality and tolerance of directionality to noise. **(A)** Calculated directionality (displacement per distance travelled) of helical paths for a long helix of curvature (*κ*) and torsion (*τ*) between 0 and 2. Directionality decreases with curvature, and increases with torsion. **(B)** Three dimensional representations of helical path shapes in the absence of curvature, with curvature but no torsion, with curvature and torsion and with curvature and high torsion. Arrows on the path indicate the orientation of the cell at that point. The corresponding curvatures and torsions are indicated in (**A**). **(C)** Simulated directionality for 10,000 swimming cells with rotation rates giving rise to path curvature (*ω*_*κ*_) and torsion (*ω*_*τ*_) between 0 and 5 rad/s, and incorporating Brownian motion. This gives the same relation as calculated directionality (**A**) and confirms simulation numerical stability. **(D)** Simulated directionality for 10,000 swimming cells with randomised *ω*_*κ*_ = 0±0 to 0±5 rad/s and *ω*_*τ*_ = 0 to 5 rad/s simulating random variation in morphogenesis affecting curvature. **(E)** Simulated directionality for 10,000 swimming cells with *ω*_*κ*_ re-randomised every 200 ms for *ω*_*κ*_ = 0±0 to 0±2 rad/s and *ω*_*τ*_ = 0 to 5 rad/s simulating random variation in flagellar propulsion.

The effect of longitudinal rotation rate on swimming behaviours can be mathematically predicted from the helical path shape generated by different longitudinal rotation rates[4,5]. The swimming path of a cell can be uniquely described by curvature, *κ*(*s*), and torsion, *τ*(*s*), of the path as functions of displacement, *s*. For a cell undergoing an unchanging swimming behaviour (omitting Brownian motion and noise through variation of propulsion or cell shape) curvature and torsion are constant. It therefore follows a helical path with constant radius, *r* = *κ*/(*κ*^2^ + *τ*^2^), and pitch, *h* = 2*πτ*/(*κ*^2^ + *τ*^2^)[6]. This can also be represented as constant angular velocity *ω*_*τ*_ perpendicular to the direction of travel and torsion as a constant angular velocity *ω*_*κ*_ parallel to the direction of travel respectively (corresponding to rotation of the Frenet-Serret frame)[41]. At low Reynolds number a reciprocating movement can only generate forward or rotational motion if it is asymmetric on time reversal[4], so the planar flagellar beat can only generate forward motion. A perfectly axially symmetric cell with axially symmetric propulsion therefore has a path curvature and torsion of zero and follows a straight line path (a helix with infinite radius and zero pitch). A break of symmetry in one dimension (such as curvature of the cell, causing asymmetric drag) introduces a rotation of the cell, causing curvature of the swimming path into a circular trajectory (a helix with zero pitch). Introduction of asymmetry in a second dimension (such as curvature of the cell in a second direction, generating a chiral cell shape) can introduce another rotation around a second axis. These two rotations curve the path into a helical trajectory, which can be calculated (see Methods). For a helical swimming path, a small path curvature yields the greatest displacement relative to distance travelled, however any increase in curvature reduces displacement (Fig 4A) and can yield futile circular paths (Fig 4B). Increasing torsion always increases displacement, and can recover the effect of high path curvature (Fig 4A). If torsion is large relative to curvature, then the radius of the helical path is small and the pitch large (the helix becomes elongated). At very high torsion to curvature ratios the helical path approaches a straight line with longitudinal rotation of the cell; a ‘twisted ribbon’ path (Fig 4B). This fundamental property of swimming paths suggests small asymmetries in cells with near axially symmetric shapes could give rise to futile circular paths, and cells may attain a strongly chiral shape to generate high path torsion, restoring highly directional swimming.

High torsion should also increase directionality for cell intrinsic sources of noise in path curvature, but not for extrinsic sources of noise like fluid flow or Brownian motion. Numerical simulation of cell swimming for *ω*_*τ*_ = 0 to 5 rad/s and *ω*_*κ*_ = 0 to 5 rad/s at 5±2 μm/s including Brownian motion (Fig 4C) recapitulates the analytical result (Fig 4A), with lower maximum directionality due to the contribution of Brownian motion. This confirms the accuracy of numerical simulation. Note for cells with *ω*_*κ*_ = 0 rad/s there was no change in directionality with *ω*_*τ*_, indicating no benefit of torsion in overcoming random translation and rotation caused by Brownian motion. Random deviation from an axially symmetric shape causing cell to cell variation in path curvature is one way intrinsic biological noise could manifest in a microorganism, through variation in cell morphogenesis. To model this, a population of cells with *ω*_*τ*_ = 0 to 5 rad/s and *ω*_*κ*_ randomly assigned per cell from 0±0 to 0±5 rad/s was simulated (Fig 4D). Increased torsion always gave increased directionality. Random variation in the flagellum beat driving motility may cause random changes in path curvature each flagellar beat, arising from noise in the molecular motors generating flagellum movement[42]. This was modelled with population of cells with *ω*_*τ*_ = 0 to 5 rad/s and *ω*_*κ*_ randomised every 200 ms per cell from 0±0 to 0±2 rad/s (Fig 4E). Again, increased torsion always increased directionality. High path torsion therefore increases directionality of swimming in the presence of path curvature and cell intrinsic noise which introduces or increases path curvature.

**Fig 5:**
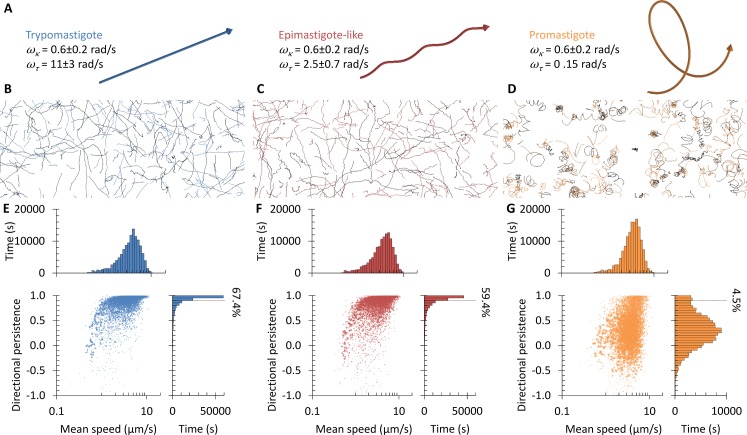
Simulation cell swimming with different speeds of longitudinal rotation captures the differences in trypomastigote, epimastigote-like and promastigote swimming. **(A)** Predicted helical path shapes for swimming trypomastigote, epimastigote-like and promastigote cells from measured longitudinal rotation rate, assuming *ω*_*κ*_ = 0.6 rad/s. **(B-D)** Paths measured from simulated low magnification videomicrographs of the simulated swimming paths of trypomastigote (**B**), epimastigote-like (**C**) and promastigote (**D**) cells for *ω*_*κ*_ = 0.6±0.2 rad/s, including the effects of Brownian motion. This simulation is based only on measured rotation rates, not the geometry of the cells. **(E-G)** Distributions and cross correlation of swimming velocity and directional persistence for simulated trypomastigote (**E**), epimastigote-like (**F**) and promastigote (**G**) cells. Point area in the scatter plots is proportional to the length of the cell track to which it corresponds.

*T*. *brucei* procyclic, *T*. *brucei* epimastigote-like and *L*. *mexicana* promastigote three dimensional swimming paths were predicted from cell longitudinal rotation rate while swimming to determine whether it could account for the differences observed. Assuming the curved cell swimming paths of *L*. *mexicana* (Fig 1B) were due to the effects of asymmetries in propulsion or drag *ω*_*κ*_ = 0.6±0.2 rad/s was selected for simulation. The measured angular velocity of longitudinal rotation were used for trypomastigote *T*. *brucei* procyclic forms (*ω*_*τ*_ = 11.0±3.0 rad/s) and *T*. *brucei* epimastigote-like procyclic forms (*ω*_*τ*_ = 2.5±0.7 rad/s) (Fig 3I). *ω*_*τ*_ = 0.15 rad/s was estimated for *L*. *mexicana* promastigotes given no longitudinal rotation was observed on time scales of 1 to 3 s. Swimming path shapes were predicted for cells swimming at 5±2 μm/s (Fig 5A). A projection into two dimensions of these helical path shapes are qualitatively similar to the experimentally determined swimming behaviours (Figs 1 and 2), and suggest that the longitudinal rotation of *T*. *brucei* is rapid enough to confer high swimming directionality, even with path curvature.

To corroborate this result, swimming of a population of cells in three dimensions (incorporating translational and rotational Brownian motion) were simulated using these angular velocity parameters. From these swimming paths simulated images, imitating low magnification videomicrographs, were generated (S2 Fig). These simulated videomicrographs were analysed by the same methods used to analyse *T*. *brucei* and *L*. *mexicana* swimming, which allowed direct comparison to the *T*. *brucei* and *L*. *mexicana* swimming data (Figs 1 and 2). This approach was taken in place of trying to infer three dimensional path shapes from the two dimensional experimental data. This simulation confirmed that the swimming paths generated for trypomastigote *T*. *brucei* (*ω*_*τ*_ = 11.0±3.0 rad/s, Fig 5B and 5E) were similar to the fast-swimming cells from experimental observations (Fig 2A and 2E). The lower path torsion (*ω*_*τ*_ = 0.15 rad/s) of promastigote *L*. *mexicana* gave a reduced capacity for highly directional swimming (Fig 5D and 5G), also similar to experimental observations (Fig 1B and 1F). The swimming paths generated by epimastigote-like *T*. *brucei* swimming (*ω*_*τ*_ = 2.5±0.7 rad/s, Fig 5C and 5F) were less highly directional than for trypomastigote *T*. *brucei*, similar to the small defect in capacity for highly directional swimming observed following induction of ClpGM6 RNAi (Fig 2B and 2D). This confirmed that the critical factor in the difference between *T*. *brucei* and *L*. *mexicana* swimming behaviours is the rate of longitudinal rotation while swimming, and that the reduction in longitudinal rotation rate of *T*. *brucei* following GlpGM6 RNAi is responsible for the reduction in swimming directionality.

### Cell shape chirality correlates with longitudinal rotation rate

*T*. *brucei* cells undergo complex movement as they swim due to the lateral attachment of the flagellum to the cell body by the FAZ. The cell presumably has an intrinsic shape defined by the organisation of sub-pellicular microtubule cytoskeleton[43], however as the cell swims this shape is constantly distorted by flagellum movement, making the effective cell shape for a swimming cell complex to determine. To determine what aspects of cell shape are chiral in *T*. *brucei* trypomastigote and epimastigote-like cells, an analytical model of the cell movement was fitted to high frame rate videomicrographs to infer the effective shape of the cell exposed to hydrodynamic drag while the cell is swimming. This is the shape of the cell with the repetitive, cyclic, movement directly due to the flagellum beat subtracted, and is different from the shape the cell would attain if the flagellum was straight and not beating or frozen at a particular stage of the beat. It includes the averaged contribution of flagellar movement on cell shape, and a tumbling cell may not have the same shape. Note that this is not a physically accurate model; instead it is a numerical description of cell shape changes as the flagellum beats which can then be used to infer three dimensional cell shape from the two dimensional source data.

**Fig 6:**
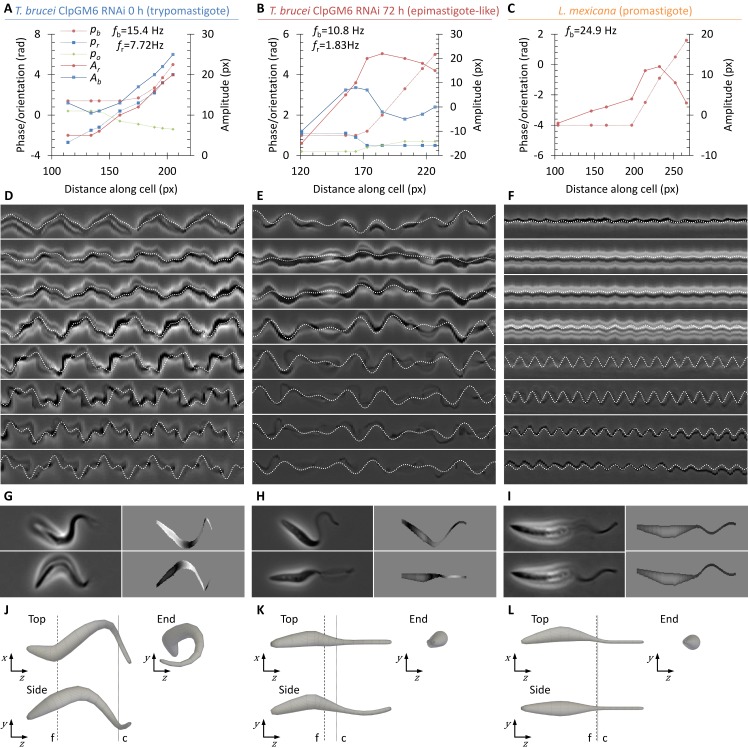
Determining the effective hydrodynamic shape of swimming promastigote, epimastigote-like and trypomastigote. **(A-C)** Fitted parameters for cell body rotation phase (*p*_*r*_) and amplitude (*A*_*r*_), flagellar beat phase (*p*_*b*_) and amplitude (*A*_*b*_) and orientation of the beat plane (*p*_*o*_) along the cell length of a representative (n = 8) trypomastigote uninduced ClpGM6 RNAi *T*. *brucei* (**A**), a representative (n = 7) epimastigote-like 72 h induced ClpGM6 RNAi *T*. *brucei* (**B**) and a promastigote *L*. *mexicana* (**C**) cell. Fitted cell rotation (*f*_*r*_) and flagellar beat (*f*_*b*_) frequencies are also indicated. **(D-F)** Example kymographs from the source high frame rate videomicrographs at different points along the cell overlaid with the movement trace from the fitted model parameters shown in (**A-C**). **(G-I)** Example still frames from the source high frame rate videomicrographs and the corresponding simulated cell appearance from the fitted model parameters shown in (**A-C**). Inferred height data is plotted through simulated phase defocus. **(J-L)** Three orthogonal views of the inferred three dimensional resting cell shape of a representative trypomastigote *T*. *brucei* (**J**), epimastigote-like *T*. *brucei* (**K**) and promastigote *L*. *mexicana* (**L**) cell. Resting cell shape was generated from the fitted model parameters shown in (**A-C**) by setting flagellum beat amplitude to zero, then rendering in three dimensions with cell body widths derived from the source high frame rate videomicrographs. Dashed lines indicate the approximate start of the flagellum (f) and end of the cell body (c).

**Fig 7:**
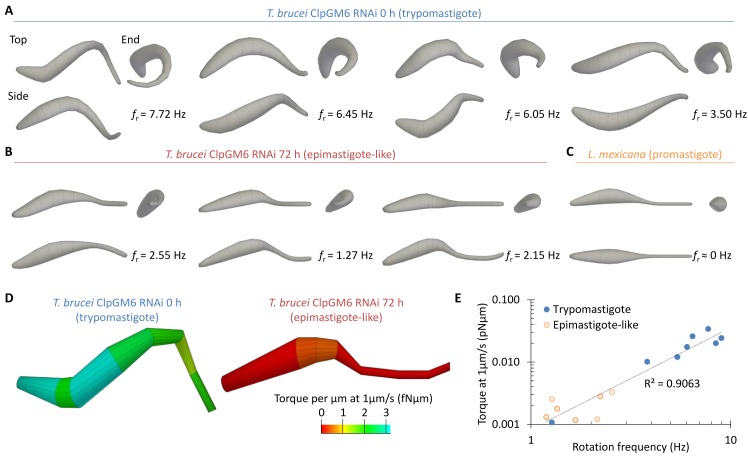
Promastigote, epimastigote-like and trypomastigote cells have increasing chirality. **(A-C)** The effective hydrodynamic shape of examples of (**A**) trypomastigote cells (uninduced ClpGM6 RNAi *T*. *brucei*), (**B**) epimastigote-like cells (72 h induced ClpGM6 RNAi *T*. *brucei*) and (**C**) a promastigote cell (*L*. *mexicana*). Frequency of cell body rotation derived from fitting is shown for each cell shape. **(D)** Torque contributing to cell rotation under 1 μm/s forward motion calculated from each segment of the effective hydrodynamic shape of a representative trypomastigote and epimastigote-like cell. More helical regions contribute more torque. **(E)** Correlation of total torque under 1 μm/s forward motion calculated from the effective hydrodynamic shape of trypomastigote and epimastigote-like cells and the frequency of cell body rotation.

The model comprises two parts: (i) A lateral displacement arising from the cell shape as it rotates longitudinally, and (ii) a lateral displacement arising from a planar sinusoid-like flagellar beat (see Methods, S3 Fig). At 8 points along the cell, values of rotation amplitude and phase, flagellar beat amplitude and phase, and orientation of the beat plane were fitted. In addition, the cell-wide values of cell rotation and flagellum beat frequencies were also fitted (Fig 6A–6C, fitting data for all cells analysed is provided in S1 Table). To confirm a plausible fit, kymographs of transverse sections through the swimming cells were compared to the fitted model of cell movement, which showed a good correspondence (Fig 6D–6F). This model allowed inference of the displacement orthogonal to the focal plane. To confirm a plausible inference of height, stills from the high framerate videomicrographs were compared with simulated stills from the model. Vertical displacement was pseudocoloured to imitate phase contrast imaging, which showed a good correspondence (Fig 6G–6I). Finally, by setting the flagellum beat amplitude to zero, three dimensional reconstructions of the effective hydrodynamic shape of the trypomastigote, epimastigote-like and promastigote cells were generated (Fig 6J–6L and Fig 7A–7C). The promastigote cell shape seems not to be chiral, but this could not be rigorously shown by this method due to the lack of longitudinal rotation of these cells. In contrast, the trypomastigote cell shape was strongly chiral, with a helical shape (Fig 6J and Fig 7A). The epimastigote-like cell shape was weakly chiral, with a twist near the cell body anterior; the short region where the flagellum is laterally attached to the cell body by the FAZ (Fig 6K and Fig 7B).

A complete three dimensional description of cell shape change over the course of a single flagellar beat is necessary for a complete hydrodynamic simulation of cell movement. This analytical model is a good approximation, but fails to capture the near-reflexive cell movement of the trypomastigote and is not physically accurate (for example it does not preserve cell length over a beat cycle). A simpler simulation based on the effective hydrodynamic shape of the cells was therefore used, treating the cell as a series of cylinders of different widths. Hydrodynamic drag on a cylinder at an angle relative to oncoming fluid flow generates a force orthogonal to the fluid flow which, if offset from the midline of the cell, causes a torque. The torque generated from drag on the effective hydrodynamic shape of trypomastigote (*n* = 8) and epimastigote-like (*n* = 7) cells was calculated assuming a fluid flow of 1 μm/s. This confirmed the most helical/chiral regions contribute most torque (Fig 7D) and showed total torque generated over the whole cell correlated well with longitudinal rotation rate (Fig 7E). During swimming this torque balances with that derived from rotational hydrodynamic drag, yielding the constant rotation rate observed.

## Discussion

This analysis of *T*. *brucei* and *L*. *mexicana* swimming showed that the swimming paths of both could be interpreted in general terms as helical paths, distorted by the effects of Brownian motion and interrupted by reversals in beat direction. This is expected for swimming in a low Reynolds number environment[4]. Trypomastigote *T*. *brucei* achieved high directionality through rapid longitudinal rotation, which can equivalently be considered as a very elongated helical path. In contrast, promastigote *L*. *mexicana* followed wider, flatter, helices with low directionality, and the epimastigote-like *T*. *brucei* mutant had intermediate behaviour. The geometry of these swimming paths confirms the established interpretation that microorganisms can be ‘spin stabilised’, demonstrating high directionality as an emergent property of the geometry of an elongated helical swimming path. Simulation of these helical swimming paths revealed an important general consequence: A microorganism can attempt to achieve highly directional swimming by two methods; either ensuring high axial symmetry to avoid any curvature of its path, or through strong chiral asymmetry to swim along an elongated helical path (which appears as rapid longitudinal rotation of the cell while swimming). Low curvature, low torsion paths were sensitive to cell intrinsic noise, with cell to cell or time dependent random change in curvature (modelling morphogenetic noise and random variation in the flagellar beat respectively) reducing directionality. This curved the swimming path giving rise to a futile circular trajectory (Fig 4). High torsion paths were insensitive to cell intrinsic noise, with increased torsion always increasing directionality, ensuring directionality of swimming (Fig 4). Understanding this phenomenon is important as very small asymmetries in shape can generate circular or low pitch helical paths (for example for sea urchin sperm[44]), highlighting the sensitivity of swimming path to cell symmetry.

The differences in helical swimming path of trypomastigote *T*. *brucei*, promastigote *L*. *mexicana* and the epimastigote-like *T*. *brucei* mutant occur despite the different cell types being of similar dimensions, with swimming driven by a flagella beat with a similar waveform. Instead, chirality in the trypomastigote and epimastigote-like cell body shape, which is not seen in the promastigote, appears responsible. In *T*. *brucei* the chirality was in the form of a helical cell shape over the portion of the cell for which the flagellum is laterally attached to the cell body by the FAZ. RNAi of ClpGM6, which shortens the FAZ[35] to generate the epimastigote-like morphology, also shortened the region for which the cell body was helical. Together these indicated that the long *T*. *brucei* FAZ is the structure responsible for cell chirality, and shows that the sub-pellicular microtubule cytoskeleton does not completely define cell shape. While *Leishmania* also have a FAZ structure[45], it is confined to the flagellar pocket and seems unable to cause shape helicity. The function of the long FAZ in generating a chiral cell shape in *T*. *brucei* is the first evidence for the function of the FAZ in swimming beyond the catastrophic failure of motility when lateral flagellum attachment is lost[35,46–52].

Most previous work concerning *T*. *brucei* swimming has considered the bloodstream form life cycle stage, also a trypomastigote. The swimming behaviours observed here for the procyclic form are similar to bloodstream forms[14,17]. The procyclic form motion in three-dimensions determined here was also similar to that determined for the bloodstream form by time-dependent tomography[16], although here cell shape could be isolated from the contribution of flagellar movement allowing calculation of hydrodynamic properties from measured cell shape. Previously, hydrodynamic simulation showed longitudinal rotation arises in a model assuming the *T*. *brucei* cell is straight and elastic, with the flagellum laterally attached with a short helical section[53]. This supports the concept demonstrated here, that longitudinal rotation emerges from chirality of cell organisation, but the cell shapes determined here suggest that assuming a straight cell and flagellum of equal length to the straightened cell may be naïve. As the trypomastigote is a morphology associated with the *Trypanosoma*-type dixenous life cycles with a stage in a vertebrate bloodstream[21], it also suggests an importance for highly directional swimming in this type of life cycle both in the vector gut and the bloodstream of the host.

The directionality of swimming achieved by *T*. *brucei* and *L*. *mexicana* may be linked to chemotaxis; *Leishmania* species undergo chemotaxis[54–57] and *T*. *brucei* undergo complex movement patterns[58,59] likely associated with chemotaxis. There are two major methods for achieving chemotaxis[60]: Firstly biased random walks; a random walk made up of directional swimming interspersed by tumbles which randomly reorientate the cell, where a high chemoattractant concentration promotes swimming (as used by *Escherichia coli*[61]). Secondly helical clinotaxis and helical path-dependent phototaxis; a processes where chemoattractant concentration or light source orientation is sampled around a curved path and modulates helical path curvature, bending the axis of the helix towards the chemoattractant/light source (as used by sperm of some metazoa[6,44] and many phototactic organisms[62]). Deterministic chemotaxis is more efficient, but for very small cells, where rotational diffusion rapidly randomises orientation, the biased random walk is more favourable[60]. The mechanism of helical clinotaxis requires a large radius helical path with directional movement achieved along the axis of the helix, whereas reliable straight line swimming is favourable for the swimming (run) stages of biased random walks. Although not tested here, it seems both modes of chemotaxis may benefit from the directionality and cell intrinsic noise tolerance conferred by path torsion. The *Leishmania* and *T*. *brucei* swimming behaviours appear well adapted for helical clinotaxis and biased random walk chemotaxis respectively. It is possible these cells are near the threshold size where, in their particular environments, biased random walk chemotaxis becomes favourable. Alternatively highly directional swimming in the absence of a chemoattractant may be beneficial for *T*. *brucei* for, for example, penetrating tissue.

By providing quantitative analysis of the phenomenon of ‘spin stabilisation’ using two human parasites, this work revealed *T*. *brucei* use a canonical example of helical cell shape to cause rapid longitudinal rotation and highly directional swimming, while *L*. *mexicana* do not. Simulation of the resulting swimming behaviours reveals a possible explanation for why swimming microorganisms often have asymmetric shape, as the longitudinal rotation caused by asymmetric drag or propulsion leads to directional swimming insensitive to noise in shape or propulsion, while a highly symmetric shape is sensitive to such noise. This provides a quantitative framework for understanding the diverse, complex and asymmetric shapes of swimming cells and microorganisms seen across the scales of microscopic life [1–3,8–10]. It is an important design criterion for artificial self-propelled microscale swimmers, and enhances the understanding of the role of cell shape in *T*. *brucei* and *L*. *mexicana* parasite swimming.

## Methods

### Cells and cell culture

Leishmania mexicana (WHO strain MNYC/BZ/62/M379) were grown in M199 supplemented with 10% FCS and 50 mM HEPES·HCl pH 7.4. Procyclic form Trypanosoma brucei (strain Lister 427) and the inducible ClpGM6 RNAi cell line[35] were grown in SDM79[63]. ClpGM6 RNAi was induced by addition of 1 μg/ml doxycycline. Cultures were grown at 28°C and maintained between 1×10^5^ and 2×10^7^ cells/ml by regular subculture, and cultures between 5×10^6^ and 1×10^7^ cells/ml were used for analysis. Culture density was measured with a CASY model TT cell counter (Roche Diagnostics).

### Motility analysis

Low magnification videomicrographs for motility analysis were captured using dark field illumination at a frame rate of 5 Hz, using a 10× NA 0.25 objective on a DM5500 upright microscope (Leica Microsystems) and an Orca CCD camera (Hamamatsu Photonics) or Neo sCMOS (Andor) with an exposure time of 2 ms. 5 μl cell culture was held in a sealed 10×10×0.25 mm volume generated with an adhesive geneframe (Fischer Scientific), a slide and a coverslip, and imaged at the ambient temperature of 26°C. Cell swimming tracks from low magnification videomicrographs and simulated low magnification videomicrographs were analysed using custom automated analysis tools written in the ImageJ[36] macro language. The minimum projection of the stack (non-moving background structures) was first subtracted from the video, then cells were identified in every frame by applying a 2 pixel (1.3 μm) Gaussian blur and finding local signal maxima. Swimming paths were built by connecting each cell location in a frame to its nearest neighbour in the previous frame based on its predicted location from the motion in the previous two frames. Tracks were built within a maximum connection distance of 15 pixels (3.2 μm). If no cell could be found within that range then the track was terminated.

Two statistics were calculated from the swimming tracks, as previously described for bloodstream form *T*. *brucei*[17], from the velocity of cells $v_{t}=\left[\begin{array}{c} x_{t+\delta t}-x_{t}\\ y_{t+\delta t}-y_{t} \end{array}\right]/\delta t$ over a time evaluation step *δt* for tracks at least 5 s long. Mean speed *s* = |***v***_*t*_| and directional persistence $d=\frac{v_{t}.v_{t+\delta t}}{\left|v_{t}||v_{t+\delta t}\right|}$ were calculated per track. Tracks were presented weighted by temporal length to eliminate bias towards cell swimming behaviours that had a tendency to generate short cell swimming paths. An evaluation step of *δt* = 2 s to assay cell swimming behaviours and omit reciprocating movement directly due to the oscillation of the flagellar beat or cell rotation was selected by determining the population mean of autocorrelation directional persistence (calculated with *δt* = 0.5 s) for autocorrelation evaluation intervals between 0 and 10 s (S1C Fig), as previously described[17].

### High frame rate videomicroscopy

High frame rate videomicrographs for analysis of cell movement resulting from the flagellar beat were captured using phase contrast illumination at a frame rate of 200 Hz, using a 100× NA 1.4 objective on a DM5500 upright microscope (Leica microsystems) and a Neo cooled sCMOS camera (Andor) with an exposure time of 5 ms. 1 μl cell culture was held in an area of approximately 10×10 mm, giving a volume depth of approximately 10 μm, and imaged at the ambient temperature of 26°C. Individual cells were cropped from the video, and swimming translation digitally subtracted assuming a constant velocity[13].

### Simulation of cell swimming and chiral stabilisation

Directionality of swimming omitting Brownian motion and biological noise was calculated assuming constant swimming speed and constant curvature and torsion giving a helical path. A helix can be defined parametrically with parameter *p* by *x* = *r* cos *p*, *y* = *r* sin *p*, *z* = *cp* giving a helix radius *r* = *κ*/(*κ*^2^ + *τ*^2^) and pitch *h* = 2*πc* = 2*πτ*/(*κ*^2^ + *τ*^2^). The length along a helical arc from *p* = 0 to *p* = *P* is given by $l=P\sqrt{r^{2}+c^{2}}$. For a single turn of the helix *P* = 2*π* giving $l=\sqrt{{\left(2\pi r\right)}^{2}+h^{2}}$. Displacement from the origin for a particular distance along the helix is the distance between the points on the helix at *p* = 0 and *p* = *P*.*δx* = *r* cos *P* − *r*, *δy* = *r* sin *P*, *δz* = *cP*, therefore displacement $s=\sqrt{{\left(r\;\cos\;P-r\right)}^{2}+{\left(r\;\sin\;P\right)}^{2}+c^{2}P^{2}}$. Substituting for *T*, *r* and *h* allows the calculation of the ratio *s*/*l* as a measure of directionality from *κ* and *τ*. In the case of large *P*, where *hP* ≫ *r*, lateral displacement *δx* and *δy* become negligible and *s* ≈ *cP*. In this case $s/l\approx c/\sqrt{r^{2}+c^{2}}=\tau /\sqrt{\kappa^{2}+\tau^{2}}$.

Directionality of swimming with Brownian motion and biological noise was calculated by numerical simulation (S2 Fig). Each cell was assigned a speed *s*, longitudinal angular velocity *ω*_*τ*_ and orthogonal rotational velocity *ω*_*κ*_, each randomised from a normal distribution with the specified standard deviation around the specified mean. Changes to cell location (vector ***l***) and orientation (orthonormal unit vectors ***t***, direction of travel and long axis of the cell, ***b*** and ***n***) by cell movement was simulated in time steps (*δt*) of 200 ms using Euler’s method and calculating rotations using quaternions: First the cell was translated by *sδt* in the direction ***t***, secondly ***t*** and ***n*** were rotated around ***b*** by *ω*_*κ*_*δt*, thirdly ***b*** and ***n*** were rotated around ***t*** by *ω*_*τ*_*δt*, then finally the cell was translated along ***t***, ***n*** and ***b*** and rotated around ***t***, ***n*** and ***b*** by randomised values to simulate Brownian motion.

For each time step translational Brownian motion was simulated as normally distributed random displacement $\sqrt{2D\delta t}$, according to the Stokes-Einstein relation[64] *D* = *kT*/6*πrμ* where *k* is the Boltzmann constant and *T* is the thermodynamic temperature (299.15 K, 26°C). The cell was modelled as a prolate spheroid *r*_*d*_ = 1.5 μm for displacement along ***t*** and *r*_*np*_ = 8.0 μm for displacement along ***n*** and ***b***. For each time step rotational Brownian motion was simulated as normally distributed random rotation $\sqrt{2fE\delta t}$, according to the Stokes-Einstein-Debye relation[65] *E* = *kT*/8*πμa*^3^ where $a=\sqrt[{3}]{{r_{d}}^{2}r_{np}}$. Rotation around each direction was adjusted according to the Perrin rotational friction coefficients *f* of a prolate spheroid[66], relative to a sphere of equal volume. The friction coefficients around the major (*f*_*d*_) and minor axes (*f*_*np*_) are given by:

$$e=\frac{r_{np}}{r_{d}}\;\xi =\frac{\sqrt{\left|e^{2}-1\right|}}{e}\;S=\frac{2\;\tanh^{-1}\xi }{\xi }$$

$$f_{d}=\frac{4}{3}\frac{\xi^{2}}{2-S/e^{2}}$$

$$f_{np}=\frac{4}{3}\frac{1/e^{2}-e^{2}}{2-S(2-1/e^{2})}$$

For calculation of swimming tolerance to sources of noise, swimming was simulated for 10,000 cells starting at the origin ($l=\left[\begin{array}{c} 0\\ 0\\ 0 \end{array}\right]$) with $t=\left[\begin{array}{c} 1\\ 0\\ 0 \end{array}\right]$ for 102.4 s, after which directionality (|***l***|/*st*) was calculated.

For simulation of dark field low magnification micrographs, swimming was simulated for 2300 cells starting at random locations in a three dimensional volume 3328×2808×100 μm with cyclic boundary conditions for 102.4 s. For comparison to experimental data, images for each time step were calculated from simulated point spread estimated using defocused *L*. *mexicana* adhered to a glass slide.

### Analytical fitting of cell movement

The three dimensional shape of the cell was determined by fitting the high frame rate videomicrographs to the following analytical model of cell shape as they swam by hand, guided by minimising *R*^2^ from traced kymographs. The coordinates used were *z* along the long axis of the cell, *x* for lateral displacement in the focal plane and *y* for vertical displacement orthogonal to the focal plane.

Displacement at any point along the cell body due to its rotation of an underlying shape is given by:

$$x_{r}(z,t)=A_{r}(z)\;\sin\left(p_{r}(z)+\omega_{r}t\right)$$

$$y_{r}(z,t)=A_{r}(z)\;\cos\left(p_{r}(z)+\omega_{r}t\right)$$

Where *A*_*r*_(*z*) and *p*_*r*_(*z*) are the amplitude and phase of cell displacement along the cell. *ω*_*r*_ is the angular velocity.

Cell and flagellum displacement due the flagellar beat were approximated as a sinusoid:

$$b(z,t)=A_{b}(z)\;\sin\left(p_{b}(z)+\omega_{b}t\right)$$

Where *A*_*b*_(*z*) and *p*_*b*_(*z*) are the amplitude and phase of movement due to the flagellar beat along the cell. *ω*_*b*_ = 2*πf*_*b*_ where *f*_*b*_ is the frequency of the flagellar beat. Non-linear *p*_*b*_(*z*) allows modelling of both travelling flagellum beat waves and rigid cell oscillations due to the flagellar beat.

The flagellar beat is planar, therefore the observed magnitude of *b*(*z*) depends on the orientation of the cell at that point along its length, given by *p*_*r*_(*z*). In addition, the orientation of the flagellum may vary relative to the orientation of the cell. The combination of displacement due to cell body rotation and movement due to the flagellar beat is therefore:

$$x_{c}(z,t)=x_{r}(z,t)+b(z,t)\;\sin\left(p_{r}(z)+p_{o}(z)+\omega_{r}t\right)$$

$$y_{c}(z,t)=y_{r}(z,t)+b(z,t)\;\cos\left(p_{r}(z)+p_{o}(z)+\omega_{r}t\right)$$

Where *p*_*o*_(*z*) is a function of *z* describing the orientation at which the flagellum beat is oriented relative to the orientation of the cell *p*_*r*_(*z*).

*x*_*c*_(*z*,*t*) was manually fitted to high frame rate videomicrograph kymographs at 8 values of *z* to estimate the *A*_*r*_(*z*), *p*_*r*_(*z*), *A*_*b*_(*z*), *p*_*b*_(*z*) and *p*_*o*_(*z*) functions, along with values of *ω*_*r*_ and *f*_*b*_. Multiple values of *p*_*r*_, *p*_*b*_ and *p*_*o*_ which fit the kymograph equally well at each *z* occur at intervals of 2*π*, therefore accurate fitting of the kymographs for intermediate *z* values by linear interpolation of the *A*_*r*_(*z*), *p*_*r*_(*z*), *A*_*b*_(*z*), *p*_*b*_(*z*) and *p*_*o*_(*z*) was used to determine their correct value. Efforts to fit cell movement to an oscillating cell orientation, implied by a bihelical model of cell movement[31], instead of constant *ω*_*r*_, were not successful.

*x*_*c*_(*z*,*t*) and *y*_*c*_(*z*,*t*) share parameters, therefore fitting of *x*_*c*_(*z*,*t*) to experimental data defines *y*_*c*_(*z*,*t*). Combined with an estimate of cell width *w*(*z*) this defines a three dimensional description of cell shape as it moves to swim. To confirm a good fit this was plotted as a phase contrast-like image in which defocus (*y*_*c*_(*z*,*t*)) is represented as a colour shift from black (in focus) through white to grey.

To determine the effective hydrodynamic shape of the cell, *A*_*b*_(*z*) was set to zero for all *z*. The resulting three dimensional shape was rendered in Blender.

### Calculation of torque from cell shape

Torque arising from hydrodynamic drag was calculated from theory of slender bodies in a Stokes flow[67,68]. Torque arises from forces perpendicular to the incoming fluid flow, acting around the midline of the cell. Perpendicular force per unit length was calculated in segments between each of the eight points defining the effective hydrodynamic shape, and multiplied by segment length and radial displacement from the cell midline to give torque.

## Supporting information

S1 Fig Cell tracking performance and evaluation interval determination. **(A)** Correlation of the number of cells detected by the automated image analysis tools from a low magnification dark field micrograph and defocus distance for live *L*. *mexicana* cells adhered to a slide. **(B)** Distribution of cell path lengths generated by the automated image analysis tools from a dark field videomicrograph captured at 5 Hz of a sample of swimming *L*. *mexicana*. **(C)** Autocorrelation of directional persistence for *L*. *mexicana* and *T*. *brucei*. Detail of autocorrelation in the first 1 s is inset on a different vertical scale. Decay of autocorrelation fell sharply over the first 0.5 s (for *T*. *brucei*) and 0.1 s (for *L*. *mexicana*) and began a steady exponential decay for both species after 1 s. An evaluation interval of 2 s was therefore used for all analysis of cell swimming paths.
(TIF)

S2 Fig Simulation of low Reynolds number spin stabilisation. **(A)** Diagram of the vectors ***l***, ***t***, ***n*** and ***b*** used to define cell location and orientation, the angular velocities *ω*_*κ*_ and *ω*_*τ*_ responsible for change in cell orientation and the speed *s* responsible for cell translocation as used for simulation of swimming. The swimming behaviours are derived from only these three parameters, and is not a hydrodynamic simulation based on cell shape. **(B)** Example three dimensional rendering of simulated swimming paths followed by cells undergoing promastigote swimming behaviours in a three dimensional volume. **(C)** Point spread function used to translate the three dimensional cell swimming paths into a simulated low magnification videomicrograph for comparison of swimming simulation to experimental data, and an example frame generated from the swimming paths shown in (**B**).
(TIF)

S3 Fig Cell movement analysis and resting cell shape inference. **(A)** Diagram representing the analysis scheme used to infer three dimensional cell shape from observations in a single focal plane. **(B,C)** Examples illustrating the contribution of different components of cell movement to the fitting of *x*_*c*_(*z*,*t*) to kymographs from uninduced (**B**) and 72 h induced (**C**) ClpGM6 RNAi *T*. *brucei*. *x*_*r*_(*z*,*t*) is the contribution of cell shape and rotation, *b*(*z*,*t*) is the contribution of the flagellar beat, *x*_*b*_(*z*,*t*) = *b*(*z*,*t*) sin(*p*_*r*_(*z*) + *p*_*o*_(*z*) + *ω*_*r*_*t*) is the contribution of the flagellar beat taking into account orientation of the flagellar beat plane and *x*_*c*_(*z*,*t*) = *x*_*r*_(*z*,*t*) + *x*_*b*_(*z*,*t*) is the combination of both cell body rotation and flagellum beating.
(TIF)

S1 Table Fitting parameters for shape determination of all trypomastigote (n = 8) and epimastigote-like (n = 7) cells. (XLSX)

S1 Video High speed videomicroscopy of promastigote, epimastigote-like and trypomastigote cell swimming. The swimming motion of **(top)** trypomastigote cells (uninduced ClpGM6 RNAi *T*. *brucei*), **(middle)** epimastigote-like cells (72 h induced ClpGM6 RNAi *T*. *brucei*) and **(bottom)** a promastigote cell (*L*. *mexicana*). Playback is at ⅛ original speed.
(AVI)
