# Supplementary figures and images for: Use of chiral cell shape to ensure highly directional swimming in trypanosomes
Source: PLoS Comput Biol. 2017 Jan 31;13(1):e1005353. doi: 10.1371/journal.pcbi.1005353 (PMC5308837; doi:10.1371/journal.pcbi.1005353)

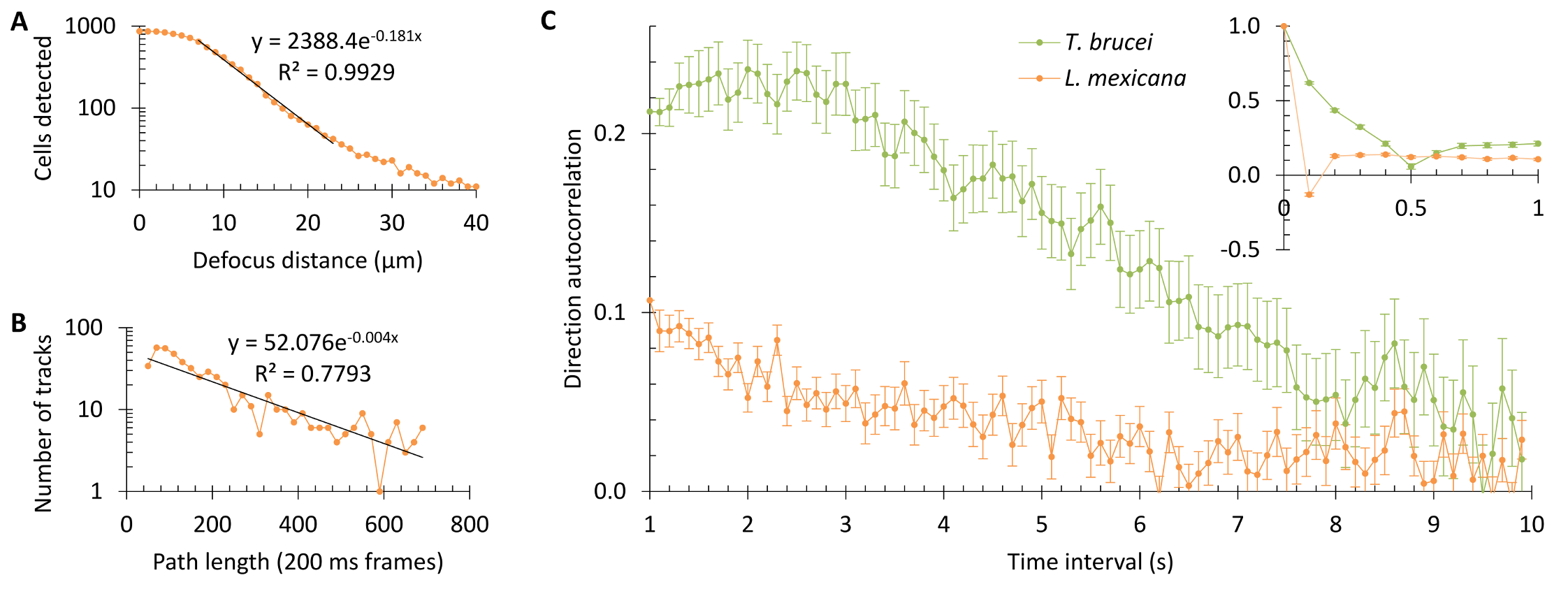

Supplement: S1 Fig — (A) Correlation of the number of cells detected by the automated image analysis tools from a low magnification dark field micrograph and defocus distance for live L. mexicana cells adhered to a slide. (B) Distribution of cell path lengths generated by the automated image analysis tools from a dark field videomicrograph captured at 5 Hz of a sample of swimming L. mexicana. (C) Autocorrelation of directional persistence for L. mexicana and T. brucei. Detail of autocorrelation in the first 1 s is inset on a different vertical scale. Decay of autocorrelation fell sharply over the first 0.5 s (for T. brucei) and 0.1 s (for L. mexicana) and began a steady exponential decay for both species after 1 s. An evaluation interval of 2 s was therefore used for all analysis of cell swimming paths. (TIF) [file pcbi.1005353.s001.tif]

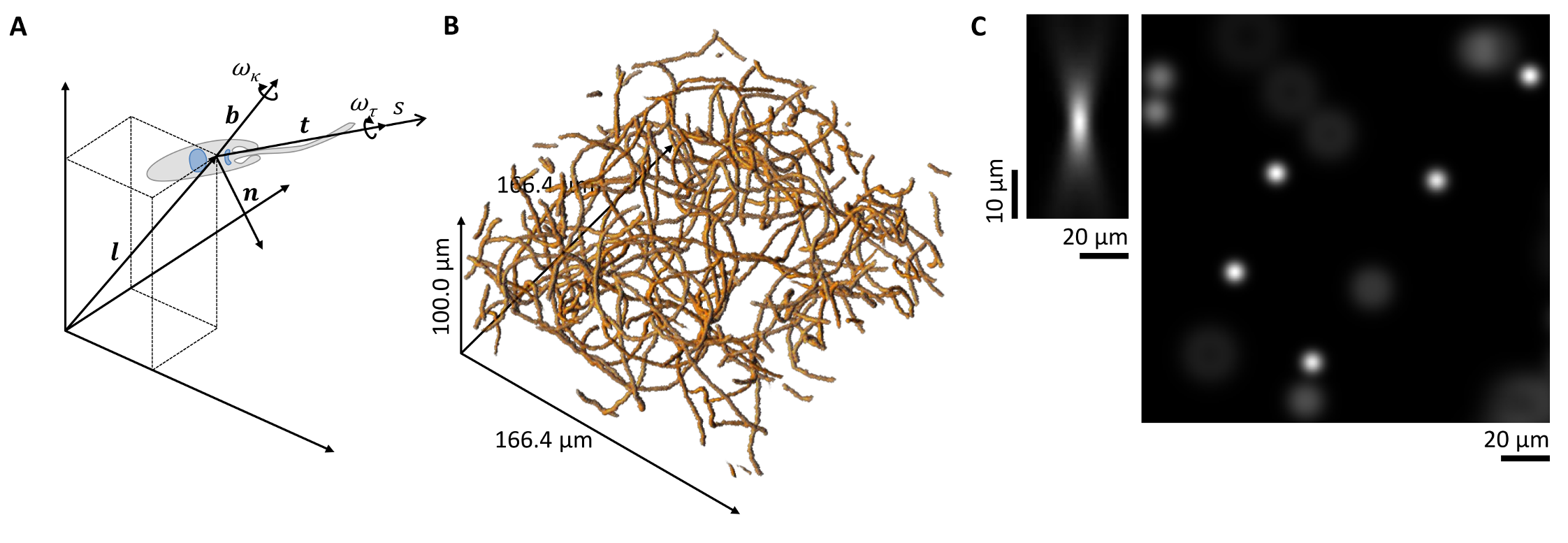

Supplement: S2 Fig — (A) Diagram of the vectors l, t, n and b used to define cell location and orientation, the angular velocities ωκ and ωτ responsible for change in cell orientation and the speed s responsible for cell translocation as used for simulation of swimming. The swimming behaviours are derived from only these three parameters, and is not a hydrodynamic simulation based on cell shape. (B) Example three dimensional rendering of simulated swimming paths followed by cells undergoing promastigote swimming behaviours in a three dimensional volume. (C) Point spread function used to translate the three dimensional cell swimming paths into a simulated low magnification videomicrograph for comparison of swimming simulation to experimental data, and an example frame generated from the swimming paths shown in (B). (TIF) [file pcbi.1005353.s002.tif]

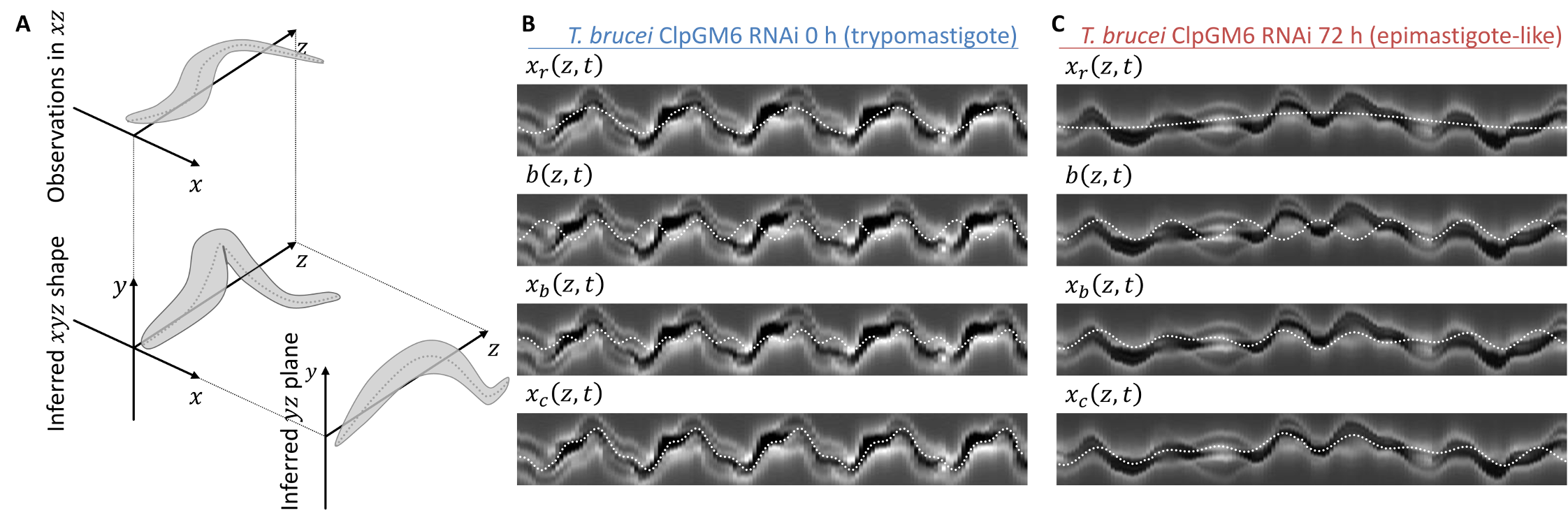

Supplement: S3 Fig — (A) Diagram representing the analysis scheme used to infer three dimensional cell shape from observations in a single focal plane. (B,C) Examples illustrating the contribution of different components of cell movement to the fitting of xc(z,t) to kymographs from uninduced (B) and 72 h induced (C) ClpGM6 RNAi T. brucei. xr(z,t) is the contribution of cell shape and rotation, b(z,t) is the contribution of the flagellar beat, xb(z,t) = b(z,t) sin(pr(z) + po(z) + ωrt) is the contribution of the flagellar beat taking into account orientation of the flagellar beat plane and xc(z,t) = xr(z,t) + xb(z,t) is the combination of both cell body rotation and flagellum beating. (TIF) [file pcbi.1005353.s003.tif]
